# A novel partial lid for mechanical defeatherers reduced aerosol dispersion during processing of avian influenza virus infected poultry

**DOI:** 10.1371/journal.pone.0216478

**Published:** 2019-05-08

**Authors:** Jianjian Wei, Jie Zhou, Yakun Liu, Jie Wu, Tao Jin, Yuguo Li, Hui-Ling Yen

**Affiliations:** 1 Institute of Refrigeration and Cryogenics/Key Laboratory of Refrigeration and Cryogenic Technology of Zhejiang Province, Zhejiang University, Hangzhou, China; 2 Department of Mechanical Engineering, The University of Hong Kong, Pokfulam, Hong Kong SAR, China; 3 School of Public Health, Li Ka Shing Faculty of Medicine, The University of Hong Kong, Hong Kong SAR, China; 4 Guangdong Provincial Center for Disease Control and Prevention, Guangzhou, China; Nanjing Agricultural University, CHINA

## Abstract

Infectious virus-laden aerosols generated during poultry processing may mediate airborne transmissions of avian influenza at live poultry markets. To develop effective control measures to reduce aerosol dispersion, we characterised the aerosol flow pattern of the mechanical defeatherers, a major source of aerosol dispersion during poultry processing at live poultry markets in China. Mechanical defeatherers create a strong air circulation during operation with inflow and outflow velocities over 1 m/s. A partial lid was designed to suppress the outflow and reduce aerosol dispersion. Computational fluid dynamics simulations confirmed that the partial lid prototype reduced the aerosol escape rate by over 65%. To validate the effectiveness of the partial lid in reducing aerosol dispersion, a field study was conducted at a retail poultry shop in Guangzhou and the concentrations of influenza viral RNA and avian 18S rRNA dispersed in air were monitored during poultry processing, with and without the use of the partial lid. At the breathing zone of the poultry worker, the use of the partial lid effectively suppressed the upward airflow and reduced the concentration of avian 18S rRNA in the air by 57%. The economic and practical partial lid can be easily implemented to reduce generation of influenza virus-laden aerosols at live poultry markets.

## Introduction

Among the multiple subtypes of avian influenza viruses (AIV) that have reportedly infected humans, H5N1, H5N6, H10N8, H7N7 and H7N9 have caused lethal infections in humans since 1997 [1–4]. H5N1 and H7N9 subtypes are of the most concern as these viruses have repeatedly caused zoonotic human infections with high case fatality rates [5,6]. The majority of human infections occurred following direct or indirect exposure to infected poultry at the human-poultry interface, including poultry farms and live-poultry markets [7].

Live poultry markets (LPMs) play a critical role in maintaining, amplifying and disseminating AIV among poultry and from poultry to humans. Previous epidemiological studies have demonstrated the transmission potential of avian influenza virus via contact, droplets and airborne routes at LPMs [8–15]. AIV may replicate in the intestinal and respiratory tracts of the infected poultry, and infectious viruses can be shed in faeces or released in exhaled breath. Highly pathogenic influenza viruses may replicate in multiple tissues and further cause systemic infections in the infected poultry, posing additional risks for processing poultry carcasses. In countries where AIV is enzootic, surveillance studies have demonstrated that genetically diverse AIV are highly prevalent among poultry at LPMs. As such, AIV are frequently detected on contaminated surfaces at LPMs [8–11]. Viral RNA or infectious AIV were also detected in the air inside LPMs [13–15] and downwind from a wholesale LPM [12]. LPMs are widely distributed in both urban and suburban areas in China, and impose a significant threat on neighbouring residents, poultry market/shop workers and visitors.

Human behaviour at LPMs may also facilitate the generation and dispersion of AIV-laden particles during the processing of AIV-infected poultry, which increases the infection risks for both poultry workers and customers. Infectious AIV have been detected in aerosols dispersed by mechanical defeatherers during poultry processing at LPMs in China [15]. In a recent study, Bertran et al. [16] experimentally demonstrated that aerosols generated during the manual processing of infected poultry mediated AIV transmission to chickens and ferrets placed within the same airspace. Most retail shops at LPMs are commonly equipped with mechanical defeatherers to process poultry for customers. The use of a mechanical defeatherer significantly increased the total number of aerosols compared to manual defeathering [17]. The motor-driven disk of the defeatherer produces strong air circulation, which is favourable for aerosol generation and dispersion. Despite its common availability and the potential risk of dispersing AIV-laden particles, there has been no study that systematically investigated the flow characteristics of the mechanical defeatherers. Such knowledge is essential to develop practical and effective control measures to reduce the risk of AIV risk at LPMs. In this study, we characterised the flow pattern and dispersion of AIV from a mechanical defeatherer using a smoke test and computational fluid dynamics (CFD). A novel partial lid was developed, and its effectiveness in reducing aerosol dispersion was validated at a LPM in Guangzhou, China.

## Materials and methods

Our studies involved laboratory flow pattern analysis, CFD simulations, and a field study. The flow pattern of a mechanical defeatherer was characterised in the building environment lab of The University of Hong Kong, and the CFD simulation was performed to further reveal the flow characteristics and quantify the reduction of aerosol dispersion using a partial lid. Finally, a field study was carried out in a poultry retail shop of a wholesale market in Guangzhou, China.

### Flow pattern characterisation by lab experiment

**Fig 1:**
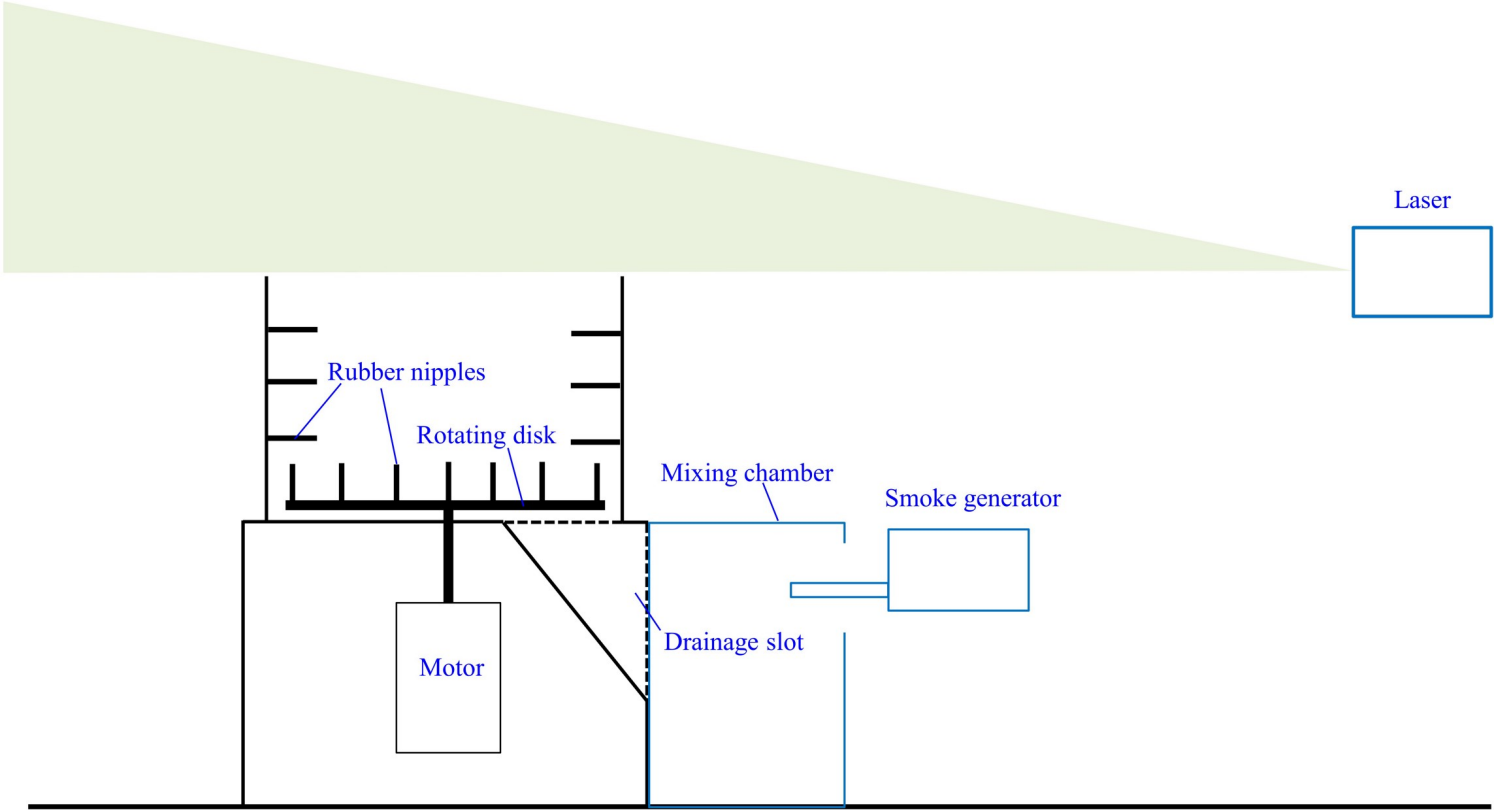
Schematic of the experiment set-up for smoke-laser visualisation (camera not shown).

The 2200 W mechanical defeatherer (MK-2200, Zhaoji, Foshan, China) had overall dimensions of 84 cm (L) × 68 cm (W) × 93 cm (H). The interior diameter of the cylindrical part was 62 cm with an opening of 54 cm. The rotating disk located at the bottom of the cylinder was driven by a motor, which ran at a speed of 185 r/min during operation. There were a number of rubber rods (10 cm in length, 2 cm in diameter) both on the cylinder wall and the disk for defeathering purposes (Fig 1). Feathers were washed away through the gap between the disk and the cylinder wall and collected at the outlet of the drainage slot.

The experiment was conducted in a room with the dimensions of 7.0 m (length) × 4.0 m (width) × 2.5 m (height). Air-conditioners were turned off to reduce disturbance. The measured background velocity was below 0.01 m/s.

A smoke generating machine (Concept Air Trace, Berks, UK) that generated fine particles below 0.5 μm was used for the visualisation experiment, as illustrated in Fig 1. The mechanical defeatherer was turned on, and smoke was released after it reached a steady state condition. As smoke from the generator has initial momentum, a mixing chamber was used as a buffer to reduce its disturbance to the air flow. The smoke out of the defeatherer was illuminated by lamps, and a black screen was used to enhance the contrast. In addition, a laser sheet was produced by a computer-controlled 3 W DPSS 532 nm laser projector (Ourslux Lighting Technology Co, Ltd) to reveal the flow dynamics. It illuminated the sagittal plane above the defeatherer as shown in Fig 1. Data were recorded using a Canon 6D camera with 24–105 lens at 25 fps.

To quantitatively explore the air flow, velocities above the cylinder were measured with a hot-sphere anemometer (AirDistSys 5000, SENSOR Electronic, Gliwice, Poland). At each point, the velocity was continuously monitored for three minutes. Three tests were carried out, and the results were averaged.

### Computer simulations using CFD

Three-dimensional models of the mechanical defeatherer with and without a partial lid were reconstructed and meshed with the aid of SolidWorks 2014 and ANSYS ICEM CFD 14.5, respectively. Flow velocity field and aerosol trajectories while the defeatherer was under operation were solved with the commercial CFD software, ANSYS Fluent 14.5. This study adopted the computing-efficiency and robust RNG k-ε model for simulating the flow [18,19]. The velocity field was obtained, and the aerosol motion equation was solved. The volume of the chicken and its rubbing with the rubber rods were not included in the simulation. For simplicity, the rubber rods of the rotating disk were treated as a uniform aerosol source. The number of released aerosols in each simulation was over 36,000 (Table 1). A humid environment in both the defeatherer cavity and surrounding room was expected. Hence, droplet evaporation was not considered. The background airflow in the room was assumed to be still.

**Table 1:** CFD-predicted reduction of escaped aerosols from the mechanical defeatherer using a partial lid.

Configuration	Aerosol diameter (μm)	No. of released aerosols	No. of escaped aerosols	Reduction by the lid
No lid	5	36,572	2,163	/
No lid	10	36,572	3,096	/
No lid	20	36,572	2,864	/
No lid	50	36,572	360	/
With a lid	5	36,410	725	66.3%
With a lid	10	36,410	1,068	65.4%
With a lid	20	36,410	714	75.0%
With a lid	50	36,410	2	99.4%

The study used a second-order upwind scheme for all the variables except pressure for the Reynolds averaged Navier-Stokes (RANS) equations, and discretisation of pressure was based on a staggered scheme PRESTO. The so-called SIMPLE algorithm was employed to couple the continuity and momentum equations. The final computational mesh contained 9.13 million tetrahedral cells for the original configuration of the mechanical defeatherer and 10.21 million tetrahedral cells for that with a partial lid after careful grid independent tests.

### Field study in a poultry retail shop

**Fig 2:**
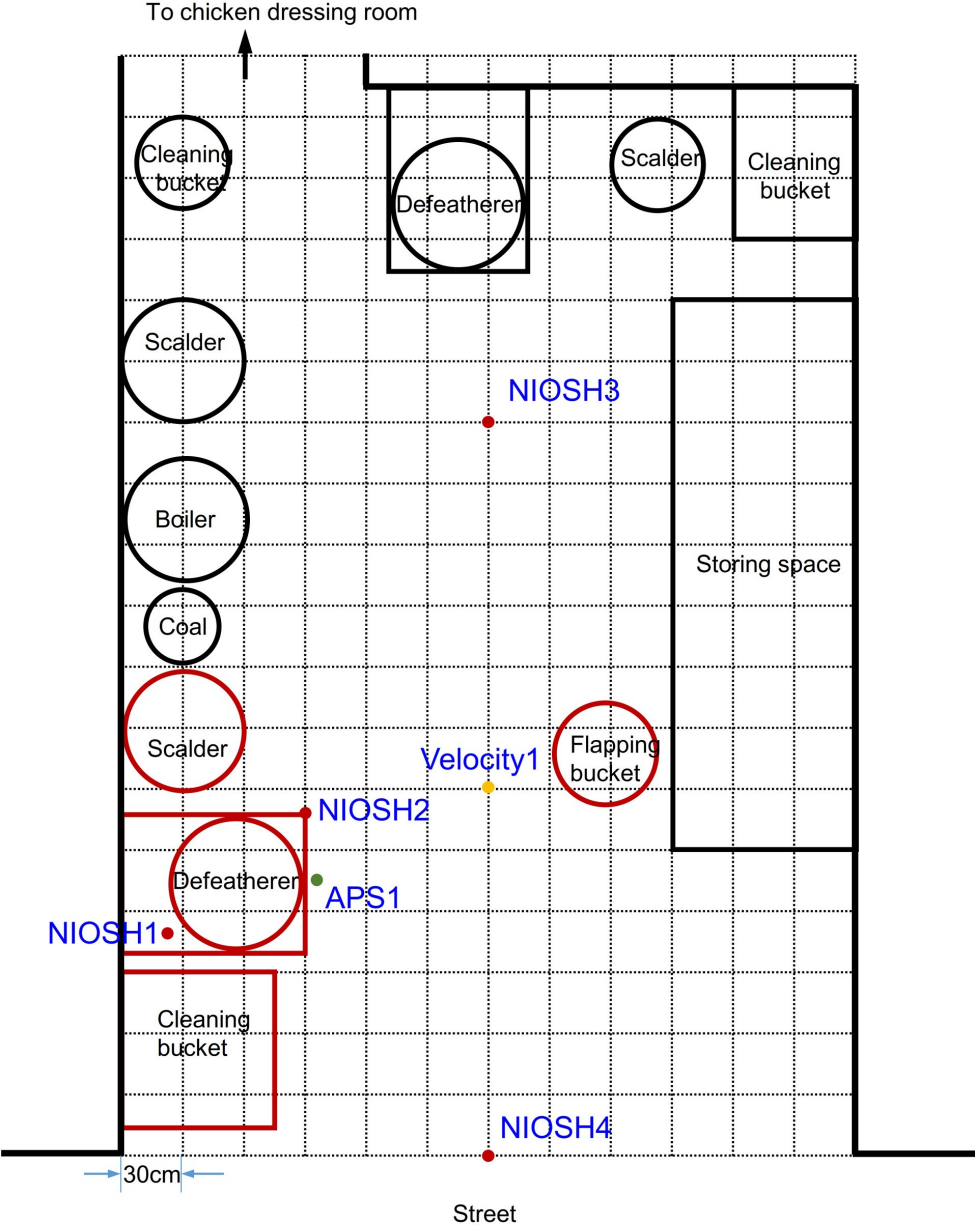
Layout of the defeathering room. The mechanical defeatherer, scalder, cleaner and flapping bucket used for the experiment are highlighted in red.

Four runs of field experiments were carried out in a poultry retail shop on 27 and 28 July 2016 in Guangzhou (Fig 2). Animal ethics approval has been obtained from The Committee on the Use of Live Animals in Teaching and Research (CULATR) at the University of Hong Kong (CULATR# 4115–16) to collect poultry swabs at live poultry markets. Poultry slaughtering and defeathering procedures were performed by the poultry shop owner while the air was sampled by our research team. In each experiment, the poultry retail shop was first ventilated for approximately 30 minutes to remove background virus-laden aerosols from the air. Afterwards, ten chickens were subjected to oropharyngeal swab sampling to monitor the level of avian influenza infection. The chickens were slaughtered by the shop owner by the severing of the jugular vein and kept inside a flapping bucket. The mechanical defeatherer was turned on, and the chickens were defeathered one by one within 30 minutes. The standard procedure for defeathering a chicken was as follows: scalding (about 40 s), defeathering in the mechanical defeatherer (about 20 s), cleaning, and dressing. Water temperature in the scalder was kept in the range 64 to 68°C during each experiment. The defeatherer was operated by a skilled poultry worker to simulate the routine poultry slaughtering process. Water was poured into the defeatherer frequently to wash away feathers during the defeathering process.

Room temperature was 32.5°C and 35.8°C on July 27 and July 28, respectively. Relative humidity was 71.0% and 55.5% on these two days, respectively. The background velocity was monitored at site Velocity1 (1.0 m and 1.6 m in height, respectively) by hot-sphere anemometers. Aerosol concentrations were measured using the Aerodynamic Particle Sizer (APS, TSI 3320) at site APS1 (6 cm away and 8 cm above the top of the mechanical defeatherer). Cyclone-based NOISH samplers (model BC251) were operated during the 30-minute defeathering process at sites NIOSH1 to 4 (NIOSH1 was 10 cm from the defeatherer and 12 cm above the cylinder top, and NIOSH2 to 4 were 1.6 m above the ground). The NIOSH samplers could collect aerosols from three size categories (i.e., > 4 μm, 1 to 4 μm, and < 1 μm) with a designated flow rate of 3.5 L/min [20]. After the experiment, 1 mL of MEM with 4% BSA was added to each of the collection tubes and PTFE filters of the NIOSH samplers to resuspend airborne particles collected from the air. Samples were analysed by real-time PCR to obtain the concentration of avian influenza virus and avian 18S rRNA, as described previously [12]. Briefly, total RNA was extracted by RNeasy Mini kit (Qiagen); AgPath-ID One-Step RT-PCR Reagents (Life Technologies) and QuantiTech SYBR Green RT-PCT reagents were used to target influenza A virus M genes and 18S rRNA, respectively.

## Results

### Visualisation and measurement of the airflow patterns of operating mechanical defeatherers

**Fig 3:**
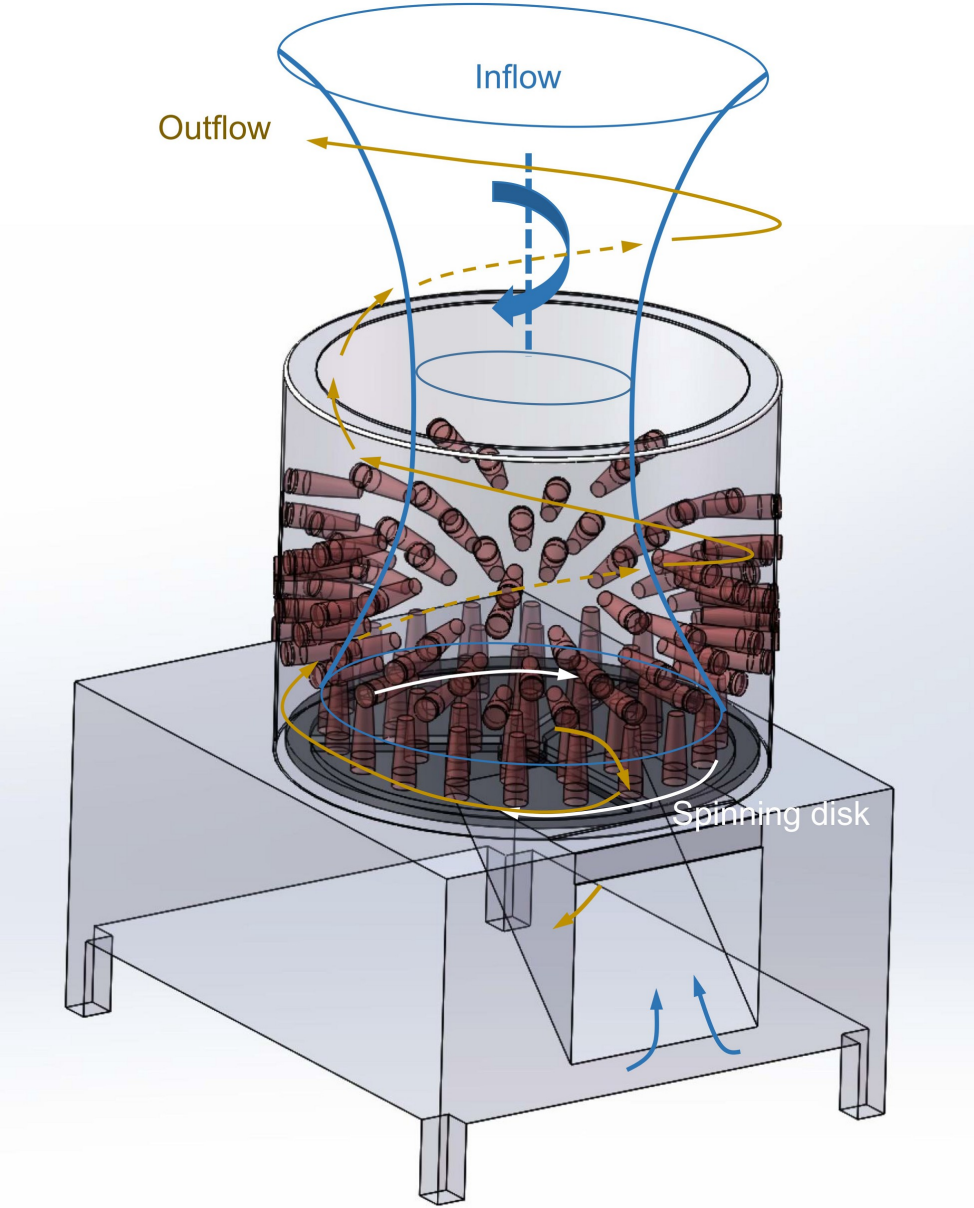
Illustration of the airflow pattern of operating mechanical defeatherers.

**Fig 4:**
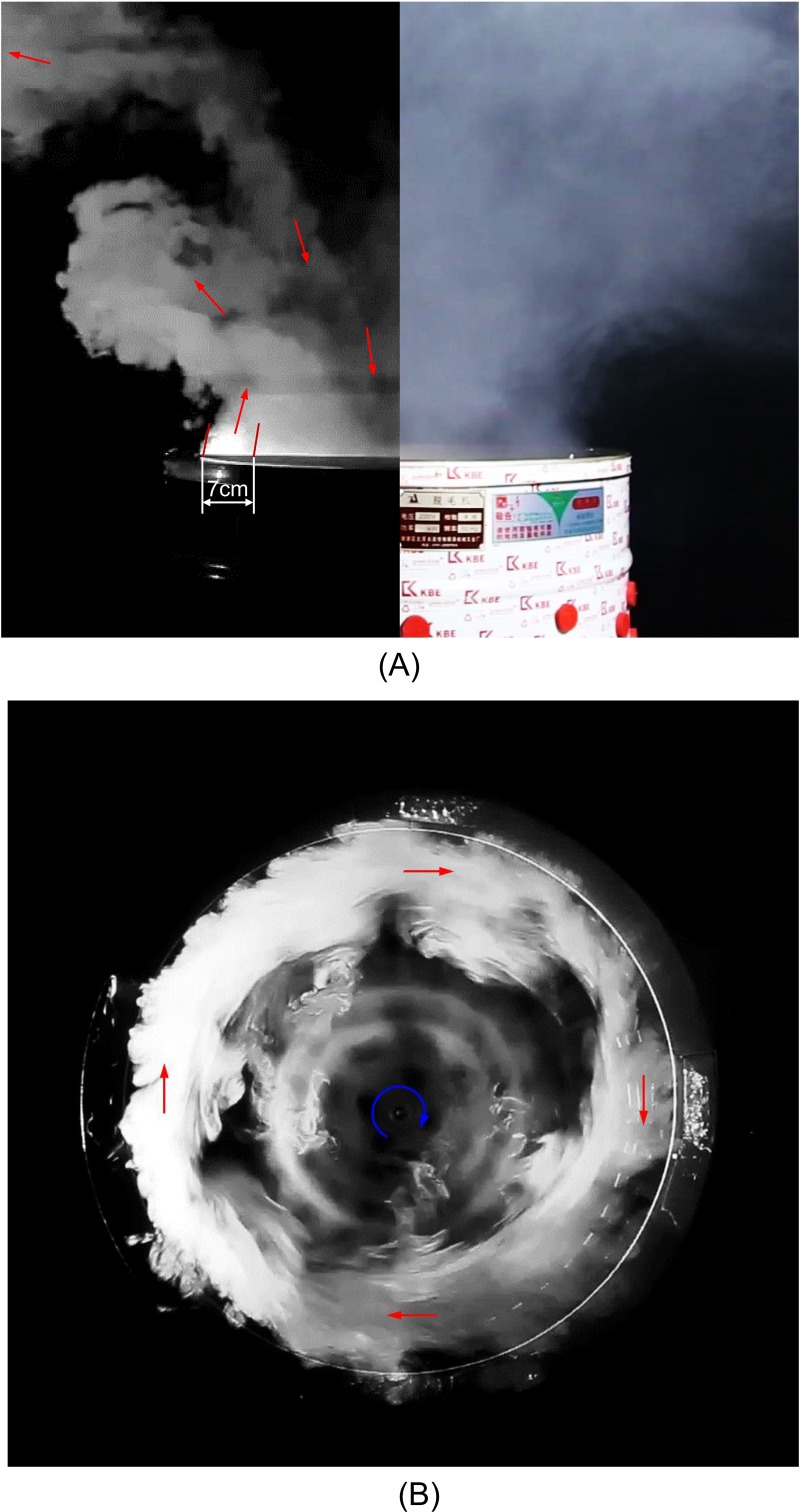
Visualisation of the air pattern above the mechanical defeatherer. (A) side view, illuminated by the laser sheet (left) or by the lamps (right) and (B) top view, illuminated by the laser sheet.

**Fig 5:**
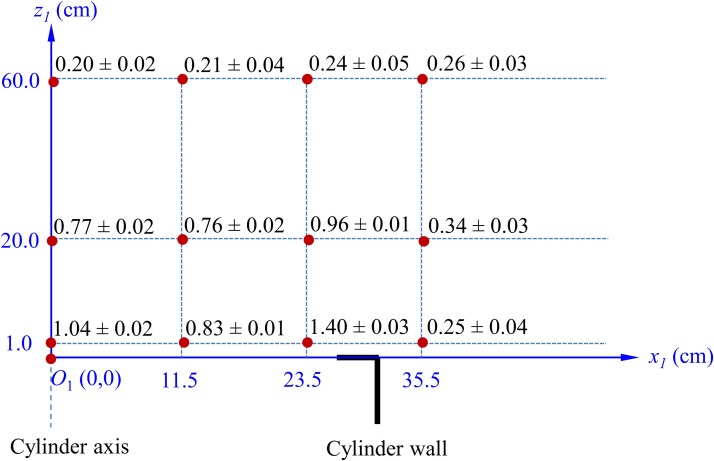
Measured velocity field above the mechanical defeatherer.

Rotation of the disk generated strong air circulation above the mechanical defeatherer. Figs 3 and 4 reveal its flow pattern (see more details in S1 Video), and the velocity magnitude is shown in Fig 5. The centrifugal effect of the rotational disk and bounding effect of the cylinder wall created an outflow that spiralled out of the defeatherer. The thickness of the annular upward air outflow from the defeatherer cylinder (see Fig 4A) was approximately 7 cm for the system that we tested. Meanwhile, there was also a downward flow into the defeatherer because the pressure above the disk centre was relatively low. The inflow spiralled towards the centre of the top opening and accelerated rapidly after it entered the cylinder. At the interface of the outflow and inflow, the large shear stress induced a strong vortex, which shed from the outflow and enhanced the horizontal spread of the air (Fig 4B). The amount of outflow from the defeatherer was considerable, and the space above it was almost filled by smoke within 10 s after the smoke was released. The smoke readily spread after it reached the ceiling.

A disk of 60 cm in diameter (see Fig 1) was rotated at a speed of 185 rpm. Therefore, the maximum air velocity near the disk was as large as 5.8 m/s. At the defeatherer-room interface, namely the very top of the defeatherer cylinder, the spiral outflow had a thickness of about 7cm, and crossed point (23.5, 1.0) cm at a speed of about 1.40 cm/s. The maximum speed of inflow was 1.04 m/s (Point *O*_1_ in Fig 5). The air velocity decayed as the distance from the defeatherer increased and was about 0.20 m/s at *z*_1_ = 60.0 cm.

### Development of a partial lid to reduce the dispersion of aerosols from an operating mechanical defeatherer

**Fig 6:**
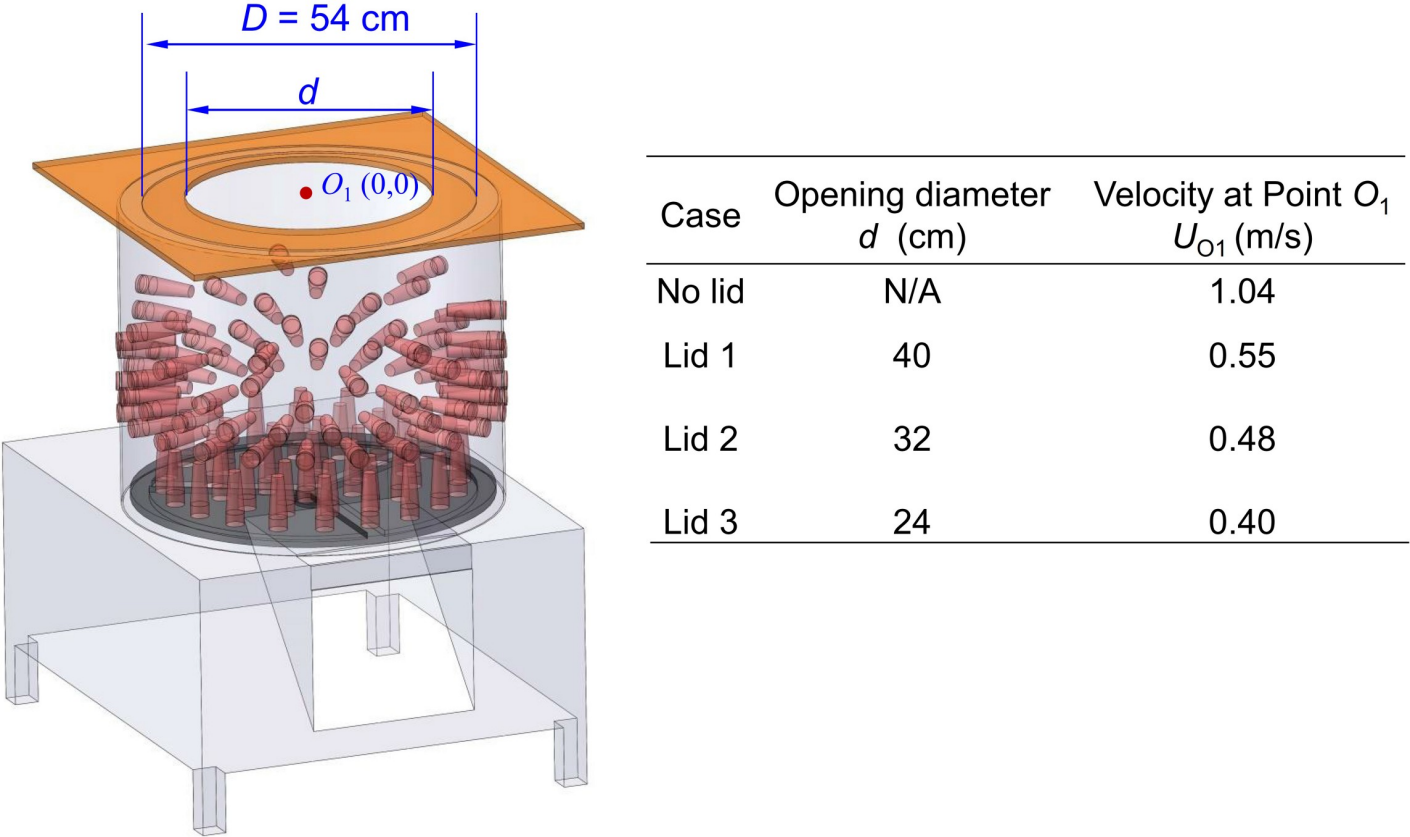
Measured velocity at the lid centre with different opening diameters.

The spiral outflow carried virus-laden aerosols. Therefore, the most direct method to reduce the spread of viruses from the mechanical defeatherer was to block the upwards air current. The thickness of the annular outflow of the defeatherer was approximately 7 cm. Thus, a partial lid made from polystyrene (0.5 cm in thickness) was added to the defeatherer cylinder (Fig 6). There was an opening in the lid to make sure that the defeathering process was not obstructed.

**Fig 7:**
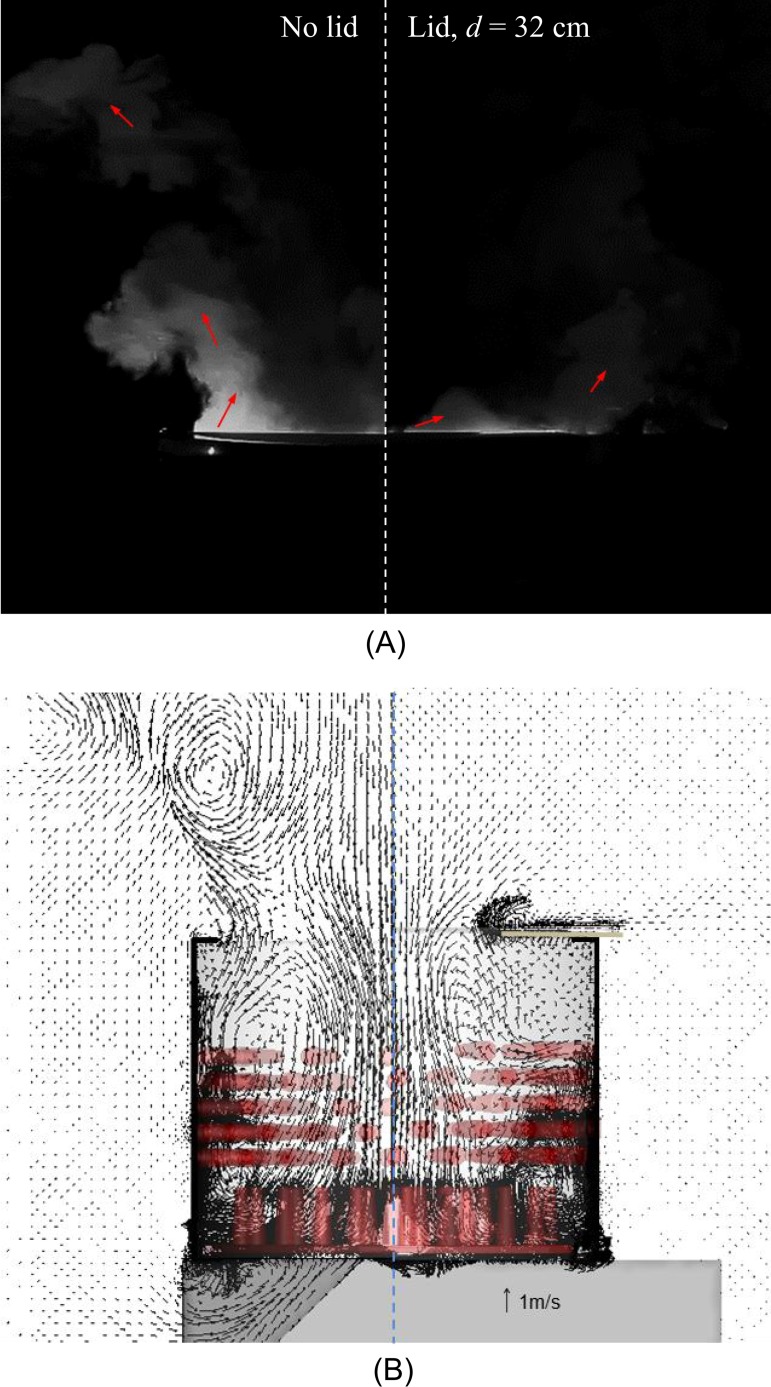
Partial lid (*d* = 32 cm) to suppress the mechanical defeatherer outflow. (A) visualisation with the laser sheet, (B) velocity vectors by CFD simulation.

Although air in the mechanical defeatherer could still escape, the effect of the lid (*d* = 32 cm; *d* is the opening diameter) was significant as shown in Fig 7A. Compared to the case without a partial lid, only a small amount of smoke was visible and did not spread upwards efficiently. Thus, the worker was much less exposed. The velocity at *O*_*1*_ was reduced to 0.48 ± 0.01 m/s, which was less than half of that without the lid. A further decrease in the cover opening (e.g., *d* = 24 cm), did not extinguish the flow circulation out of the cylinder; the velocity at *O*_*1*_ was measured to be 0.40 ± 0.03 m/s. The opening of 24 cm in diameter was relatively small for a poultry worker to operate the defeatherer. Therefore, a lid with a 32-cm opening was chosen for the CFD simulations.

**Fig 8:**
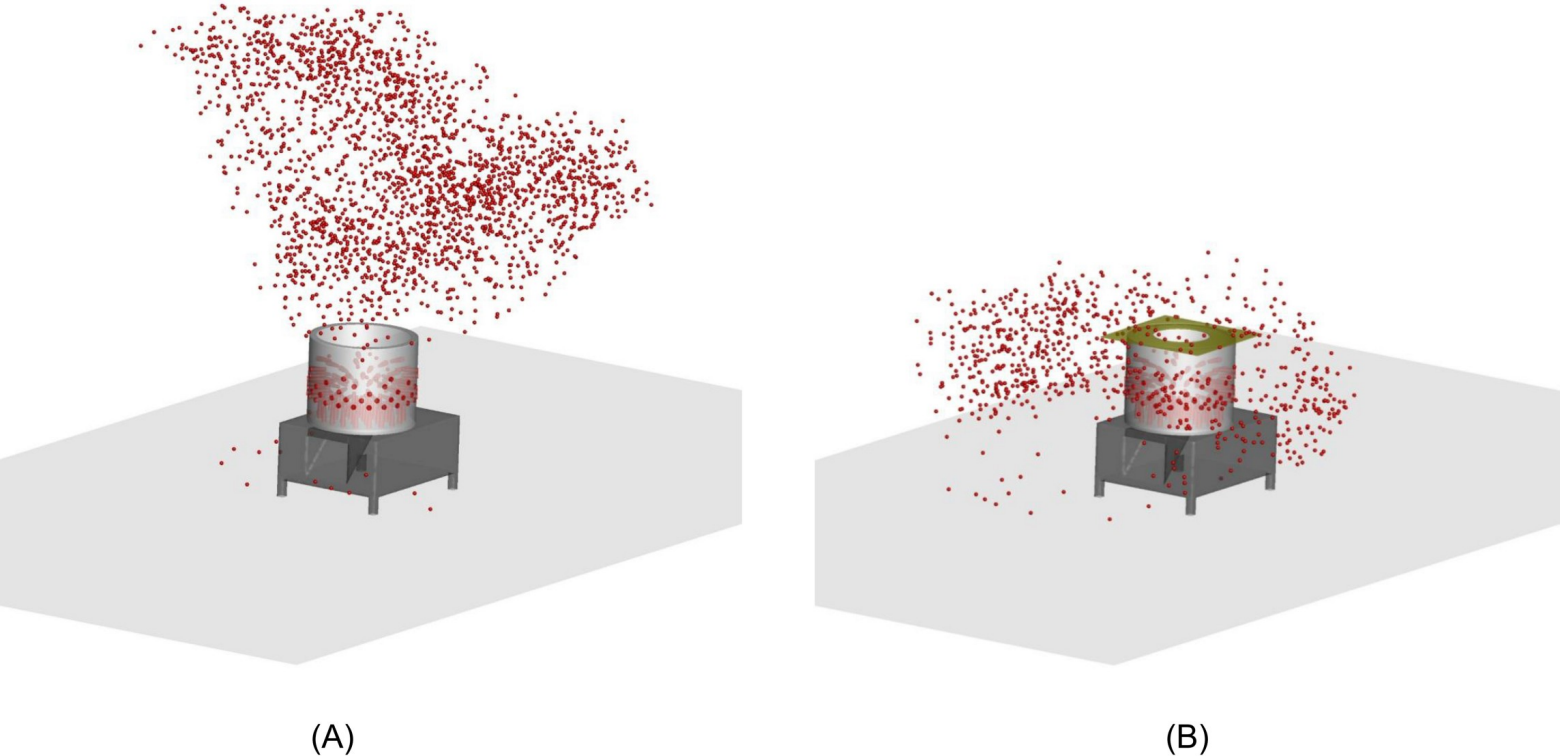
Aerosol (5 μm in diameter) dispersion from the mechanical defeatherer. (A) without or (B) with a partial lid (*d* = 32 cm).

The CFD simulations further confirmed the effect of the partial lid on the suppression of airflow (Fig 7B). In particular, the outflow that carried the virus-laden aerosols tended to escape from the mechanical defeatherer and travel horizontally (Fig 8). As a result, the partial lid efficiently prevented particle-laden air travelling to the breathing zone of the poultry workers. The partial lid also reduced the quantity of escaped aerosols. Table 2 shows the statistical results of the aerosols released from the rubber rods of the rotating disk. Taking the 5-μm aerosols as an example, 2.163 out of 36,572 aerosols escaped from the top opening of the defeatherer. In contrast, 725 out of 36,410 escaped when the partial lid was attached, resulting in a reduction rate of 66.3%. The partial lid reduced the 10-μm aerosol escape rate by 65.4%, 20-μm aerosol escape rate by 75.0%, and almost contained 50-μm aerosols.

**Table 2:** Air sampling results from the defeathering experiments.

Experiment No.	Use of the lid	Number of AIV positive chickens (mean ± SD, viral RNA copy number)	Concentrations of AIV M gene sampled in air (copies/m^3^)				
			Size	NIOSH1	NIOSH2	NIOSH3	NIOSH4
1	No	1/10 (1,224 ± 4,569)	≤ 1 μm	Neg*	Neg	Neg	Neg
			1–4 μm	3.79×10^2^	Neg	Neg	Neg
			≥ 4 μm	Neg	5.41×10^3^	1.28×10^3^	4.41×10^2^
2	Yes	5/10 (4,004 ± 6,445)	≤ 1 μm	8.33×10^2^	Neg	Neg	Neg
			1–4 μm	Neg	Neg	Neg	7.02×10^2^
			≥ 4 μm	1.34×10^4^	1.65×10^3^	1.06×10^4^	8.65×10^3^
3	Yes	4/10 (20,531 ± 41,753)	≤ 1 µm	Neg	Neg	Neg	Neg
			1–4 μm	Neg	Neg	Neg	3.85×10^4^
			≥ 4 μm	5.61×10^3^	2.23×10^3^	Neg	1.15×10^4^
4	No	4/10 (56,402 ± 103,813)	≤ 1 μm	Neg	Neg	Neg	Neg
			1–4 μm	Neg	Neg	Neg	1.49×10^4^
			≥ 4 μm	1.51×10^4^	9.18×10^3^	4.77×10^3^	8.91×10^3^

*Neg = Negative

### A field study to validate the effect of the partial lid at a retail poultry shop

**Fig 9:**
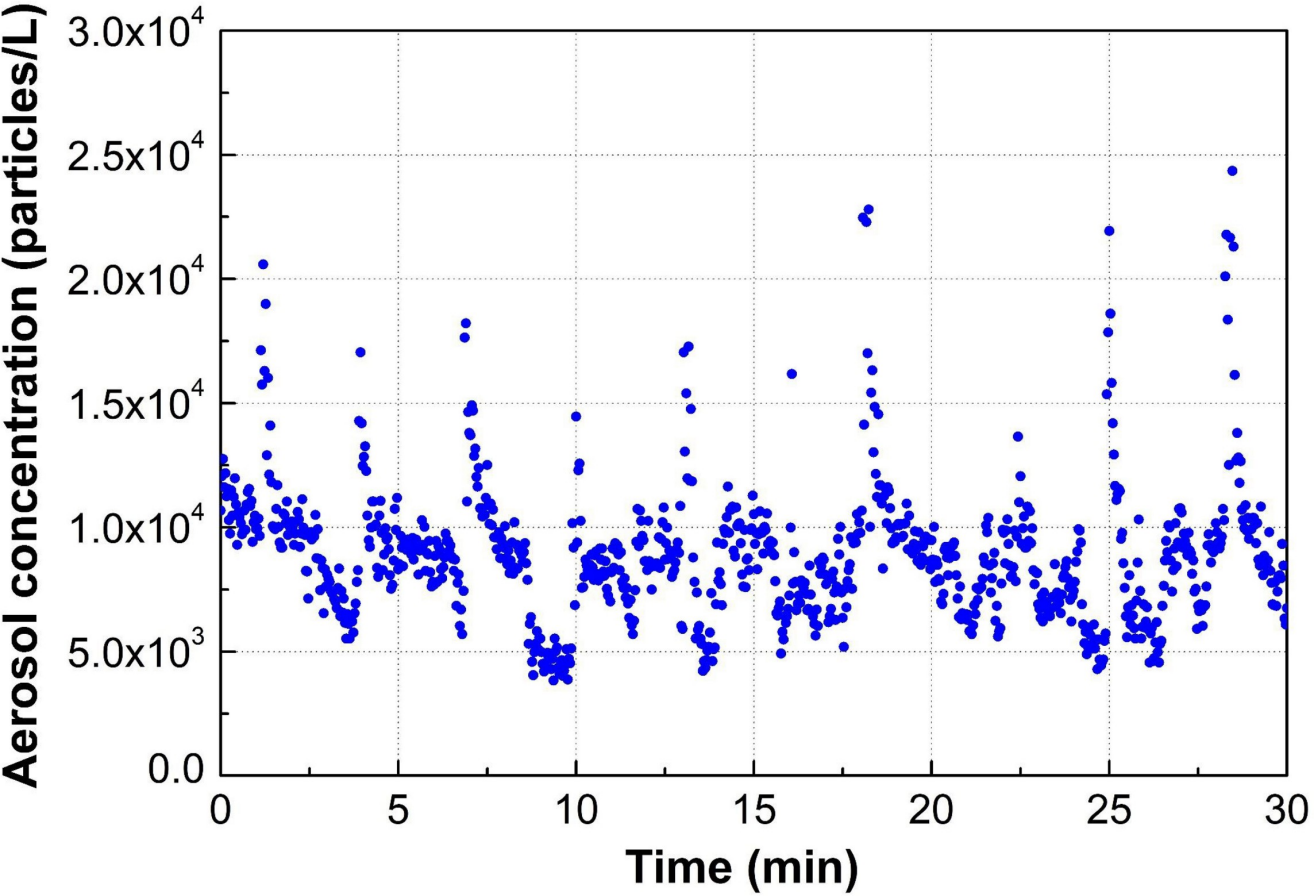
Concentration of aerosols (> 0.523 μm) during the defeathering process.

The effect of the partial lid on the release of airborne particles containing AIV viral RNA or avian 18S rRNA was evaluated at a retail poultry shop. Forty chickens (10 chickens per experiment run) were purchased from the same poultry supplier in the early mornings of 27 and 28 July 2016. AIV was detected from the oropharyngeal swab of the chickens by RT-PCR, at the positive rate of 10%-50% (Table 2). The result is in agreement to our longitudinal surveillance data collected at the LPM where this field study was performed, as we expected that approximately 39.4% of the chickens (N = 1,801) would be infected with AIV [21]. The AIV positive rate and the viral load in the poultry swab samples was the lowest in the 1^st^ experiment. The highest AIV viral load was detected in the 4^th^ experiment but only from four out of ten chickens. APS was applied to monitor the quantity of aerosol release during the experimental runs. The strong airflow of the operating mechanical defeatherer facilitated the dispersion of aerosols into the room (Fig 9). The aerosol concentration measured at site APS1 during the 30- minute run of experiment No. 1 was 3.8 ×10^3^ to 2.44×10^4^ particles/L. The aerosol concentrations increased sharply when one chicken was thrown into the defeatherer and dropped after the 20 s defeathering process. Ten sequential peaks of the aerosol concentration were recorded, which corresponded to the defeathering process of 10 chickens during the experimental run. The result corroborates the role of mechanical defeatherers as important sources for aerosol generation at the LPMs.

AIV viral RNA was readily detected in 15 out of 16 air samples collected by the NIOSH bioaerosol sampler, which separated the aerosols into three size fractions (Table 2). The AIV M gene was predominantly detected in aerosols of ≥ 4 μm (14/16), less frequently in aerosols of 1 to 4 μm (4/16), and rarely in submicron sized aerosols (1/16). The highest virus concentration was detected at 5.0×10^4^ copies/m^3^ (Site NIOSH4, Experiment 3). Concentrations of AIV viral RNA detected in air varied between experiments, possibly due to the variable AIV positive rates detected among the chickens used for the four experimental runs (Table 2).

**Fig 10:**
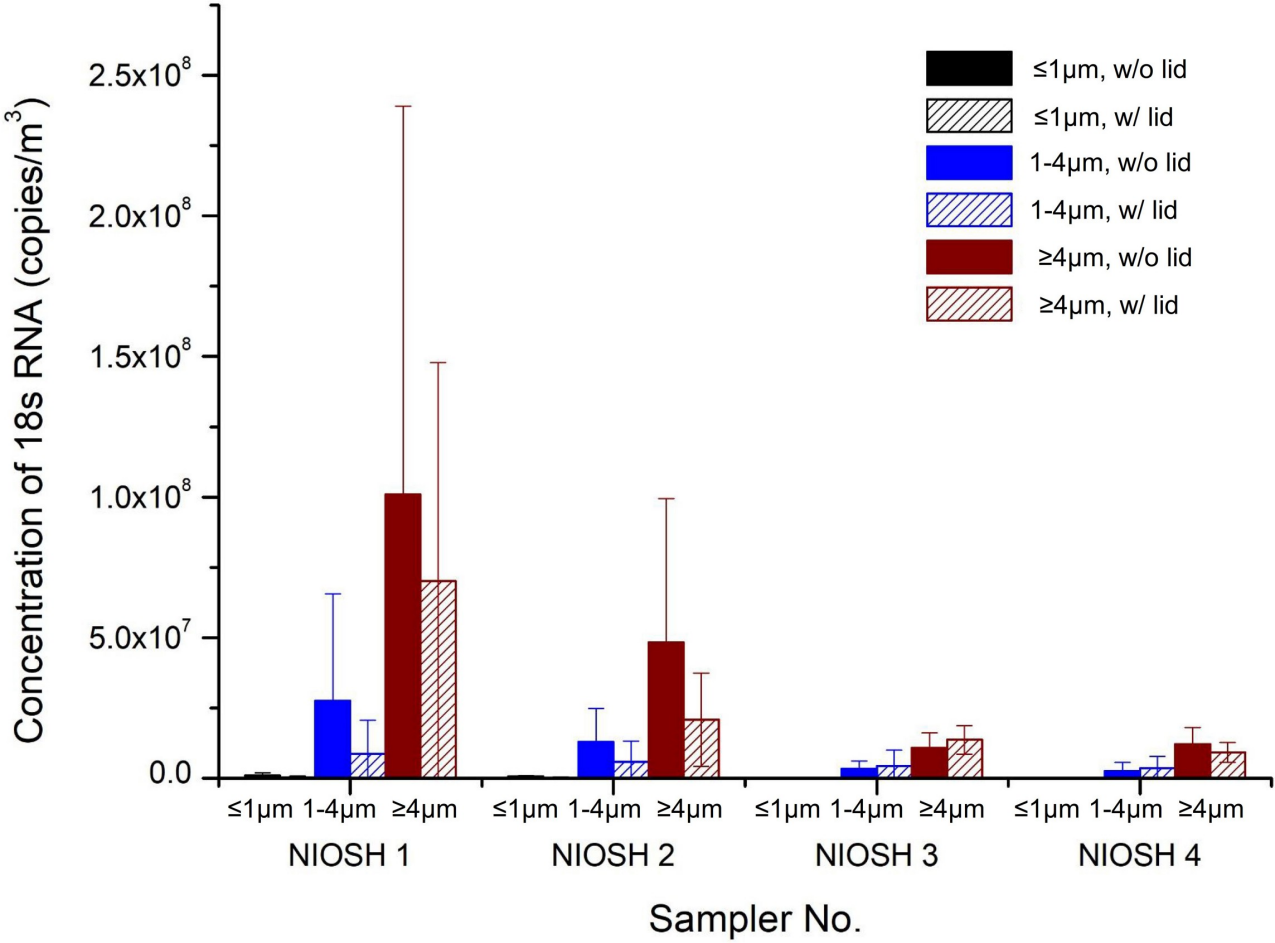
Comparison of avian 18S rRNA concentrations without or with the use of a partial lid (*d* = 32 cm).

Because not every chicken used for the field study was infected with AIV, it might not be ideal to evaluate the performance of the partial lid by comparing the AIV M gene quantity dispersed in the air. Therefore, we further quantified the avian 18S rRNA copy number in air as a surrogate ]12] to test the control effect of the partial lid. Avian 18S rRNA was a suitable housekeeping gene for quantifying genetic materials of avian species. The highest concentration of 18S rRNA in the air was detected in the 4^th^ experiment as 2.5×10^8^ copies/m^3^ at site NIOSH1 where the air sampler was immersed in the outflow of the mechanical defeatherer. It is followed by 1.1×10^8^ copies/m^3^ at site NIOSH2. The poultry worker stood close to the defeatherer during the operation and was readily exposed to AIV if there is any. With the use of a partial lid (*d* = 32 cm), the 18S rRNA concentration at the poultry worker’s breathing zone (site NIOSH2) was reduced by 57% (Fig 10; see more details in S1 Table), which was different from the simulated aerosol dispersion pattern in Fig 8B that indicated that the 18S rRNA-laden aerosols in the field study were able to reach the breathing zone due to the background ventilation. Before the use of the partial lid, 18S rRNA concentrations at site NIOSH 3 and NIOSH 4 were much lower compared to those at site NIOSH 1 and NIOSH2 because they were located relatively far from the defeatherer. However, the level of 18S rRNA reduction in the air with a partial lid was not significant at sites NIOSH3 and NIOSH4.

## Discussion

The mechanical defeatherer operates with a rotating disk and removes poultry feathers by fixed rubber rods. This process is efficient in producing relatively large aerosols. Viral RNA and 18S rRNA were predominantly detected in aerosols larger than 4 µm. Consistent with our results, the simulated slaughter of infected poultry generated viable virus predominantly in droplets (> 4 µm) and aerosols (1 to 4 µm), but not in particles (<1 µm) [1]. The highest concentration of viral RNA detected in the air at the poultry stall was 5.0×10^3^ copies/m^3^, which was two orders of magnitude lower than that detected inside the LPM (4.4×10^5^ copies/m^3^), where 10,000 to 20,000 live poultry were held daily [12]. Although we did not quantify the infectious dose of AIV in the air, viral RNA detected at 5.0×10^3^ copies/m^3^ in the air may still pose a significant AIV infection risk for poultry workers and nearby customers. It is also expected that during outbreak conditions when most of poultry are infected with AIV, the quantity of AIV-laden aerosols in air may be further increased.

**Fig 11:**
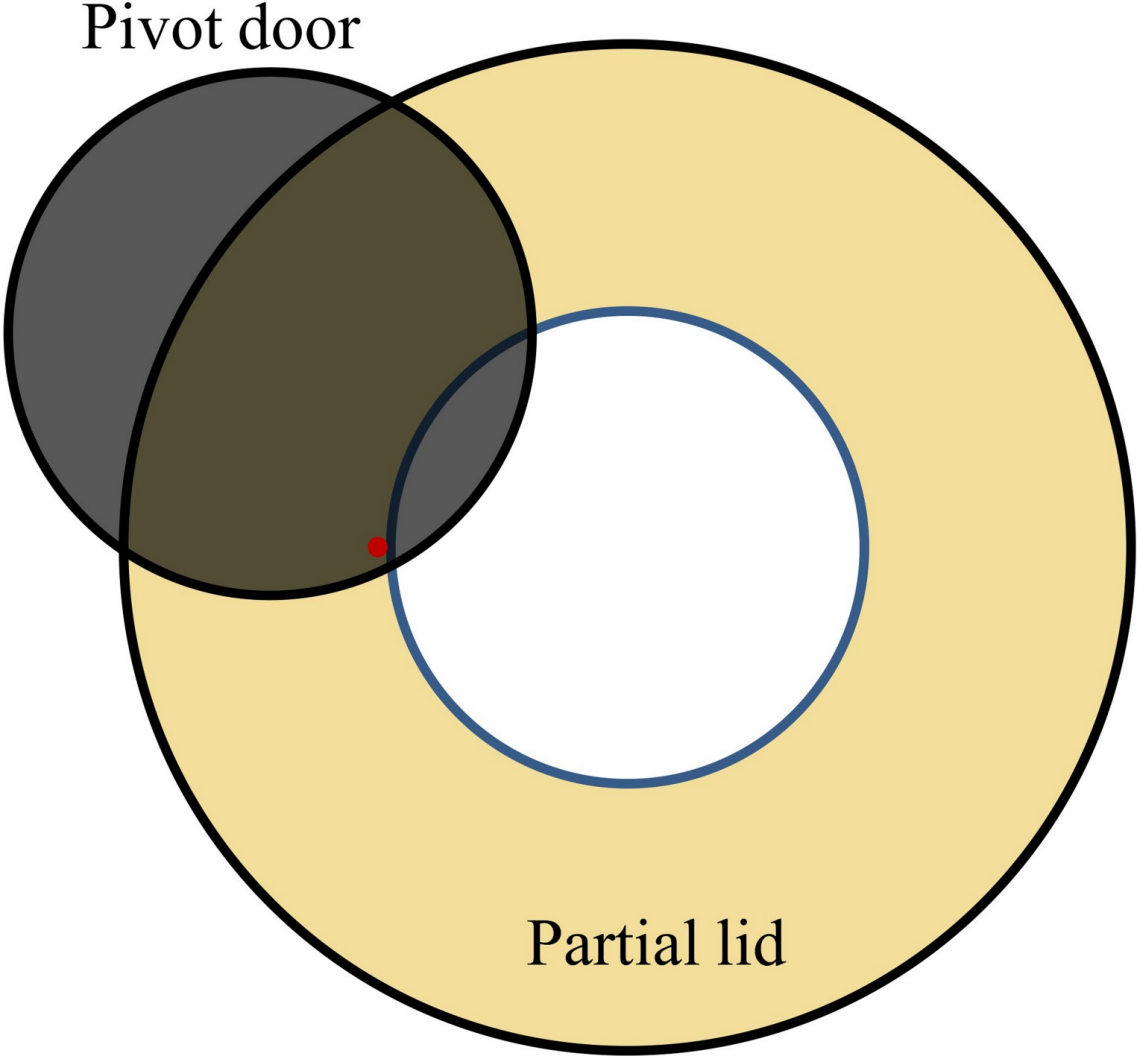
The pivot door superimposed on the current partial lid.

With the use of a partial lid, the exposure risk to the poultry worker was effectively reduced. It was not practical to completely seal the top of the mechanical defeatherer to contain the generated aerosols because poultry workers need to pour water in regularly during the defeathering process to wash away the feathers. To further reduce aerosol dispersion, one possible improvement is to add a pivot door to the opening of the current partial lid (Fig 11). The use of the pivot door would allow the poultry workers to easily open and cover the mechanical defeatherer while needed. The partial lid can be made at low cost with polystyrene, plastic or steel, can be easily installed for existing mechanical defeatherers, and provides a practical method to reduce the dispersion of virus-laden aerosols. It is worth mentioning that the concentration of influenza viral RNA or avian 18S rRNA at sites NIOSH3 and NIOSH4 were not greatly affected by the partial lid (see Fig 10 and Table 2). The partial lid effectively suppressed upward outflow, but the aerosol generation by rubbing of poultry and rubber rods was barely affected. As a result, the aerosol concentration inside the mechanical defeatherer and within the outflow may increase correspondingly. In addition, the partial lid potentiated the release of horizontal air flow (see Figs 7 and 8), which might have contributed to the increased detection of influenza virus-laden or avian 18S rRNA-laden aerosols at sites NIOSH3 and NIOSH4 while the lid was in use. However, with the addition of the pivot door, it is expected that the horizontal air flow will be effectively reduced.

During our experiment, we demonstrated that the drainage slots could draw air from the room into the mechanical defeatherer. Meanwhile, aerosols may also escape from the drainage slot (Fig 8). With the use of a partial lid, our CFD simulations indicated that the escaped aerosol numbers from the drainage slot were less than 29 and 56, respectively, for the original configuration and that with a partial lid. Methods to prevent aerosol escape from the drainage slot will be tested in a future study. Furthermore, it may be possible to refine the shape or geometry of the partial lid to further reduce both upward and horizontal aerosol dispersion. Before the live poultry markets are replaced by centralized poultry slaughterhouses, the application of simple interventions to reduce aerosol dispersion of mechanical defeatherers will help to reduce the transmission risk of avian influenza viruses.

## Conclusions

The flow pattern above the mechanical defeatherer is characterised by strong downward and upward spiral flows with velocities recorded over 1 m/s. The machine efficiently produces and disperses aerosols that carry avian influenza virus into the ambient air. Viral RNA was readily detected in 15 out of 16 air sampling events with the highest concentration recorded at 5.0×10^3^ copies/m^3^. Viral RNA and avian 18S rRNA were predominantly detected in aerosols larger than 4 µm. The partial lid prototype that we designed suppressed the release of the upward airflow from the mechanical defeatherer while potentiated the release of horizontal airflow. The partial lid reduced the aerosol escape rate by over 65% according to the CFD simulations and may offer partial protection for poultry workers by reducing their potential exposure to aerosolised AIV by 57%. Our detailed analyses of the aerosol dispersion pattern support the rationale of the lid design, which can be easily implemented to reduce dispersion of influenza-laden aerosols during poultry processing at live poultry markets.

## Supporting information

S1 Video Visualization of the defeathering machine’s flow. The machine was first started, and smoke was released after it reached the steady condition.
(MP4)

S1 Table Air sampling results of 18S rRNA from the defeathering experiments. (XLSX)

## References

[1] Chan PKS. Outbreak of avian influenza A (H5N1) virus infection in Hong Kong in 1997. Clin Infect Dis. 2002; 34: S58–S64 10.1086/338820 11938498

[2] Chen Y, Liang W, Yang S, Wu N, Gao H, Sheng J, et al Human infections with the emerging avian influenza A H7N9 virus from wet market poultry: clinical analysis and characterisation of viral genome. Lancet. 2013; 381: 1916–1925. 10.1016/S0140-6736(13)60903-4 23623390 PMC7134567

[3] Peiris JSM, Cowling BJ, Wu JT, Feng L, Guan Y, Yu H, et al Interventions to reduce zoonotic and pandemic risks from avian influenza in Asia. Lancet Infect Dis. 2016; 16: 252–258. 10.1016/S1473-3099(15)00502-2 26654122 PMC5479702

[4] Su S, Bi Y, Wong G, Gray GC, Gao GF, Li S. Epidemiology, evolution, and recent outbreaks of avian influenza virus in China. J Virol. 2015; 89: 8671–8676. 10.1128/JVI.01034-15 26063419 PMC4524075

[5] World Health Organization (WHO). Cumulative number of confirmed human cases of avian influenza A (H5N1) reported to WHO [accessed April 26, 2018]. 2018a. Available from: http://www.who.int/influenza/human_animal_interface/2018_03_02_tableH5N1.pdf?ua=1&ua=1

[6] World Health Organization (WHO). Influenza at the human-animal interface: summary and assessment, 26 January to 2 March 2018. 2018b. Available from: http://www.who.int/influenza/human_animal_interface/Influenza_Summary_IRA_HA_interface_02_03_2018.pdf?ua=1

[7] Lai S, Qin Y, Cowling BJ, Ren X, Wardrop NA, Glibert M, et al Global epidemiology of avian influenza A H5N1 virus infection in humans, 1997–2015: a systematic review of individual case data. Lancet Infect Dis. 2016; 16: e108–e118. 10.1016/S1473-3099(16)00153-5 27211899 PMC4933299

[8] Choi YK, Ozaki H, Webby RJ, Webster RG, Peiris JS, Poon L, et al Continuing evolution of H9N2 influenza viruses in Southeastern China. J Virol. 2004; 78: 8609–8614. 10.1128/JVI.78.16.8609-8614.2004 15280470 PMC479067

[9] Nguyen DC, Uyeki TM, Jadhao S, Maines T, Shaw M, Matsuoka Y, et al Isolation and characterization of avian influenza viruses, including highly pathogenic H5N1 from poultry in live bird markets in Hanoi, Vietnam, in 2001. J Virol. 2005; 79: 4201–12. 10.1128/JVI.79.7.4201-4212.2005 15767421 PMC1061558

[10] Lau EH, Leung YH, Zhang LJ, Cowling BJ, Mak SP, Guan Y, et al Effect of interventions on influenza A (H9N2) isolation in Hong Kong’s live poultry markets, 1999–2005. Emerg Infect Dis. 2007; 13: 1340–7. 10.3201/eid1309.061549 18252105

[11] Garber L, Voelker L, Hill G, Rodriguez J. Description of live poultry markets in the United States and factors associated with repeated presence of H5/H7 low-pathogenicity avian influenza virus. Avian Dis. 2007; 51: 417–20 10.1637/7571-033106R.1 17494597

[12] Wei J, Zhou J, Cheng K, Wu J, Zhong Z, Song Y, et al Assessing the risk of downwind spread of avian influenza virus via airborne particles from an urban wholesale poultry market. Building and Environment. 2018; 127: 120–126 10.1016/j.buildenv.2017.10.037 29479134 PMC5822749

[13] Chen PS, Lin CK, Tsai FT, Yang CY, Lee CH, Liao YS, et al Quantification of airborne influenza and avian influenza virus in a wet poultry market using a filter/real-time qPCR method. Aerosol Sci Technol. 2009; 43: 290–297

[14] Swayne DE. Avian influenza, John Wiley & Sons, 2009

[15] Zhou J, Wu J, Zeng X, Huang G, Zou L, Song Y, et al Isolation of H5N6, H7N9 and H9N2 avian influenza a viruses from air sampled at live poultry markets in China, 2014 and 2015. Eurosurveillance. 2016; 21: 30331 10.2807/1560-7917.ES.2016.21.35.30331 PMC5015459 27608369

[16] Bertran K, Balzli C, Kwon YK, Tumpey TM, Clark A, Swayne D. Airborne transmission of highly pathogenic influenza virus during processing of infected poultry. Emerg Infect Dis. 2017; 23: 1806–1814 10.3201/eid2311.170672 29047426 PMC5652435

[17] Bertran K, Clark A, Swayne DE. Mitigation strategies to reduce the generation and transmission of airborne highly pathogenic avian influenza virus particles during processing of infected poultry. Int J Hyg Environ Health. 2018; 221: 93–900 10.1016/j.ijheh.2018.05.013 29891217

[18] Zhang Z, Chen X, Mazumdar S, Zhang T, Chen Q. Experimental and numerical investigation of airflow and contaminanttransport in an airliner cabin mockup. Building and Environment. 2009; 44: 85–94

[19] Cao SJ, Meyers J. Influence of turbulent boundary conditions on RANS simulations of pollutant dispersion in mechanically ventilated enclosures with translational slot Reynolds number. Building and Environment. 2013; 59: 397–407

[20] Lindsley WG, Schmechel D, Chen BT. A two-stage cyclone using microcentrifuge tubes for personal bioaerosol sampling. J Environ Monit. 2006; 8: 1136–1142 10.1039/b609083d 17075620

[21] Cheng KL. Monitoring temporal changes in prevalence and viral load of avian influenza viruses at live poultry markets in Guangzhou, China, 2015–2018. Master of Philosophy (MPhil) thesis at The University of Hong Kong, 2018

